# Refeeding improves blood glucose and gastric emptying responses to a mixed-nutrient meal in anorexia nervosa

**DOI:** 10.1186/2050-2974-3-S1-O64

**Published:** 2015-11-23

**Authors:** Gabriella Heruc, Tanya Little, Michael Kohn, Sloane Madden, Simon Clarke, Michael Horowitz, Christine Feinle-Bisset

**Affiliations:** 1 University of Adelaide, Adelaide, Australia; 2 The Children's Hospital at Westmead, Sydney, Australia; 3 Westmead Hospital, Sydney, Australia

## 

Gastric emptying (GE) is an important determinant of postprandial blood glucose (BG), yet GE is delayed in anorexia nervosa (AN). This study aimed to characterise relationships between GE (13C-octanoate breath test), and BG responses to, a mixed-nutrient semi-solid meal in 22 female adolescent AN inpatients on admission (BL) and following 1 (W1) and 2 weeks (W2) of refeeding, and in 17 age-matched healthy controls (HC). Compared with HC, BL GE was markedly delayed in AN (BL:192±21, HC:310±40%/hr, P<0.01). At W2, GE was faster (W2:297±34), and no longer different from HC. Fasting BG did not differ between AN and HC, however, BG did not rise postprandially in AN at BL (BL:635±14, HC:803±29 mmol/L.min-1, P<0.01). At W2, BG increased postprandially in AN, yet remained lower than in HC (W2:713±18, P<0.05). There was a moderate correlation between GE and BG in HC (R2=0.643, P<0.01), but not in AN. In conclusion, GE of, and BG response to, a mixed-nutrient meal are markedly impaired in untreated AN, and nutritional rehabilitation may partially restore the gut responses to nutrients. This study highlights the need to elucidate the mechanisms underlying altered postprandial BG responses in AN and the importance of clinical monitoring of BG during refeeding.

